# Content comparison and person-centeredness of standards for quality improvement in cardiovascular health care

**DOI:** 10.1371/journal.pone.0244874

**Published:** 2021-01-07

**Authors:** Beatrix Algurén, Tomas Jernberg, Peter Vasko, Melissa Selb, Michaela Coenen

**Affiliations:** 1 Faculty of Education, Department of Food and Nutrition, and Sport Science, University of Gothenburg, Gothenburg, Sweden; 2 The Jönköping Academy for Improvement of Health and Welfare, School of Health Sciences, Jönköping University, Jönköping, Sweden; 3 Department of Clinical Sciences, Danderyd University Hospital, Karolinska Institute, Stockholm, Sweden; 4 Department of Internal Medicine, Central Hospital, Växjö, Sweden; 5 ICF Research Branch, a cooperation partner within the WHO Collaborating Centre for the Family of International Classifications (at DIMDI), Nottwil, Switzerland; 6 Swiss Paraplegic Research, Nottwil, Switzerland; 7 Department of Medical Information Processing, Biometry and Epidemiology—IBE, Chair of Public Health and Health Services Research, Research Unit for Biopsychosocial Health, Ludwig-Maximilians-Universität (LMU) Munich, Munich, Germany; 8 Pettenkofer School of Public Health (PSPH), Munich, Germany; Maastricht University Medical Center, NETHERLANDS

## Abstract

**Background:**

Quality standards are important for improving health care by providing compelling evidence for best practice. High quality person-centered health care requires information on patients' experience of disease and of functioning in daily life.

**Objective:**

To analyze and compare the content of five Swedish National Quality Registries (NQRs) and two standard sets of the International Consortium of Health Outcomes Measurement (ICHOM) related to cardiovascular diseases.

**Materials and methods:**

An analysis of 2588 variables (= data items) of five NQRs—the Swedish Registry of Congenital Heart Disease, Swedish Cardiac Arrest Registry, Swedish Catheter Ablation Registry, Swedish Heart Failure Registry, SWEDEHEART (including four sub-registries) and two ICHOM standard sets–the Heart Failure Standard Set and the Coronary Artery Disease Standard Set. According to the name and definition of each variable, the variables were mapped to Donabedian’s quality criteria, whereby identifying whether they capture health care processes or structures or patients’ health outcomes. Health outcomes were further analyzed whether they were clinician- or patient-reported and whether they capture patients’ physiological functions, anatomical structures or activities and participation.

**Results:**

In total, 606 variables addressed process quality criteria (31%), 58 structure quality criteria (3%) and 760 outcome quality criteria (38%). Of the outcomes reported, 85% were reported by clinicians and 15% by patients. Outcome variables addressed mainly ‘Body functions’ (n = 392, 55%) or diseases (n = 209, 29%). Two percent of all documented data captured patients’ lived experience of disease and their daily activities and participation (n = 51, 3% of all variables).

**Conclusions:**

Quality standards in the cardiovascular field focus predominately on processes (e.g. treatment) and on body functions-related outcomes. Less attention is given to patients’ lived experience of disease and their daily activities and participation. The results can serve as a starting-point for harmonizing data and developing a common person-centered quality indicator set.

## Introduction

Quality, safety and the efficiency of health care systems and public health strategies are strongly related to effectively capturing and managing information [1,2]. Since the 1990s, the European Parliament has been calling for effective health information systems and the development of harmonized health indicators as a first step [3]. In the last decade, the use of large national registries and quality standard sets has shown to be important for improving health care globally by providing compelling evidence for best practice.

The core idea behind high quality value-based health care is that patients should receive interventions that primarily aim to improve their quality of life and to prolong their life [4]. The paradigm shift towards person-centered value-based health care, in which a person’s experience of disease and of functioning in daily life are valued, was highlighted in the report *Crossing the Quality Chasm* from the Institute of Medicine (IOM; now called National Academy of Medicine) [5]. The quality of health care, as proposed by Donabedian, is defined by the interplay between changes in an individual’s functioning as a result of health care (i.e. outcomes), factors like equipment and human resources that affect the context in which care is delivered (i.e. structures), and actions taken within a health care system like treatment and other interventions (i.e. processes) [6]. This means that neither information on processes, nor on outcomes nor on structures alone is sufficient to define health care quality. Donabedian highlights medical care as a process in which all parameters and their dynamic interplay are important to understand how health care quality can be achieved [6,7]. However, the paradigm shift towards person-centered health care makes outcomes that are valued by patients and describe patients’ functioning more important than ever [8]. The importance of person-centered outcomes was underscored by Meyer et al., who proposed a quality measurement policy that supports more quality measures focusing on what matters to patients, e.g. patients lived experience of disease [9].

While health care quality measures have improved since the publication of IOM report on health care quality in 2001, these measures are still “disorganized, inefficient, confusing and misleading” [10, p.1979]. This unstructured way of documenting health information can make sharing and comparing data difficult and hinder effective decision-making and efforts toward improving health care quality, management and research. Through harmonization and standardization of health information, data sharing is possible, and in turn, can reduce the otherwise big burden of data collection and documentation on clinicians [10,11]. Given the value of harmonization and standardization of health information, many national and international projects aimed at developing a common health information system have been initiated in recent years, e.g. the Digital Health Initiative program from the United States Agency for International Development (USAID) (www.usaid.gov), the European Union project ‘Bridging Information and Data Generation for Evidence-based Health policy and Research (BRIDGE Health) [12], or the Health Data Collaborative as an informal partnership of international agencies, governments, academics and other donators (www.healthdatacollaborative.org).

A step toward harmonization and specifically standardization of health information is understanding what health information is and is not available. Content comparison of existing quality standards can contribute to a better understanding about what information is captured in those quality standards—whether the quality standards have overlapping content and can be harmonized, whether they maintain a balance between different types of information, e.g. data on processes and outcomes, and whether they consider the aspects of functioning that are important to patients. Data on both processes and outcomes would shed light on whether existing standards are person-centered, reflect the value-based health care paradigm and contain the necessary information to be able to understand the process of achieving health care quality. An in-depth examination of the content of quality standards could inform ongoing efforts toward health data harmonization, and contribute to improving quality standards as well as promote person-centered health care. To our knowledge, there are currently no studies that have examined the content of quality standards on such a detailed level.

The objective of the present study was to provide a case in point, and analyze and compare the content of quality standards, specifically five Swedish National Quality Registries (NQRs) including four sub-registries (www.kvalitetsregister.se) and two standard sets of the International Consortium of Health Outcomes Measurement (ICHOM) (www.ichom.org) related to cardiovascular diseases (CVDs). We chose to examine CVDs as they are among the main contributors to the global burden of disease [13].

## Materials and methods

### Data and study sample

In Sweden, more than 100 NQRs exist. These NQRs are valuable tools for improving health care [14,15]. With strong international collaboration and the vision of creating value-based health care, ICHOM has advanced the development of “global standard sets of outcome measures that matter most to patients”.

The study data comprising 2588 variables (Table 1) were derived from freely available variable-lists of five cardiovascular NQRs (including four sub-registries) and two ICHOM standard sets (ICHOM-SS) [16,17]. The variable lists were downloaded from the respective websites in 2017. The quality standard sets encompassed the two ICHOM-SS – for heart failure and for coronary artery disease (www.ichom.org) [16,17]. The following NQRs (Table 1) were included in the study sample: Swedish Registry of Congenital Heart Disease (SWEDCON), Swedish Cardiac Arrest Registry, Swedish Catheter Ablation Registry, Swedish Heart Failure Registry (SwedeHF), Swedish Websystem for Enhancement and Development of Evidence-based care in heart disease (SWEDEHEART). The latter comprises several sub-registries: the registries for a) acute coronary care (RiksHIA), b) secondary prevention (SEPHIA), c) heart surgery (HKIR & TAVI) and d) coronary angiography and percutaneous coronary intervention (SCAAR). SWEDEHEART was established in 1991, and many studies based on the data from the registry or its sub-registries have been published in leading international cardiovascular journals.

**Table 1:** National quality registries and ICHOM standard sets included in the content analysis.

Name	Abbreviation	Established in year	Classifications and patient-reported outcome measures (PROM) included	Total number of variables*	Total number of variables for analysis**
**Swedish National Quality Registries (NQRs)** ^**1**^					
Swedish Cardiac Arrest Registry		1990/2005	EQ-5D, HADS	247	196
Swedish Heart Failure Registry	SwedeHF	2003	EQ-5D	191	162
The Swedish Catheter Ablation Registry		2004		53	53
Swedish Registry of Congenital Heart Disease	SWEDCON	1998	EQ-5D, Disabkids, NYHA	966	623
Swedish Websystem for Enhancement and Development of Evidence-based care in heart disease (exist of several part-registries, see below)	SWEDEHEART	2008	EQ-5D, VAS-Pain, NYHA	982	806
Acute coronary care	*Common & RiksHIA*			209	180
Heart surgery	*HKIR & TAVI*			334	269
Coronary angiography and percutaneous coronary intervention	*SCAAR & CT*			319	251
Secondary prevention	*SEPHIA*			120	106
**ICHOM standard sets (ICHOM-SS)** ^**2**^					
ICHOM Coronary Arterial Disease Database		2015	SAQ-7, PHQ-2, Rose Dyspnea Scale	100	100
ICHOM Heart Failure Database		2016	KCCQ-12, NYHA, PHQ-2, PROMIS	49	49
**TOTAL**	** **	** **	** **	**2588**	**1989**

ICHOM = International Consortium for Health Outcomes Measurement; ICF = International Classification of Functioning, Disability and Health; ICD = International Classification of Diseases; EQ-5D = EuroQoL-5D; HADS = Hospital Anxiety and Depression Scale; NYHA = New York Heart Association Functional Classification; SAQ-7 = Seattle Angina Questionnaire; PHQ-2 = Patient Health Questionnaire; KCCQ-12 = Kansas City Cardiomyopathy Questionnaire; PROMIS = Patient-Reported Outcome Measurement Information System

1) Available from www.kvalitetsregister.se

2) Available from www.ichom.org

* According to the list of variables as provided on the homepage in 2017

** The number of variables after having deleted duplicate variables, i.e. repeated measurements of the same variable at different time points.

### Analysis

Relying on the framework of Donabedian’s quality criteria, i.e. outcomes (changes in an individual’s functioning as a result of health care), infrastructure/structures (factors that affect the context in which care is delivered,), and processes (actions done within health care) [6], we mapped the variables to the respective quality criteria. Duplicated variables in the NQRs (i.e. variables with identical content appearing more than once, such as blood pressure measured at different time points, n = 599) were counted only once. Variables not covered by the quality criteria were categorized as “others”. These variables were administrative (e.g., patient’s address, time point assessed) or pertaining to patient characteristics (e.g., patient’s age). There were some variables in the NQRs that had a variable code but no definition; these variables were categorized as “not defined”.

In order to investigate the person-centeredness of quality standards, the variables that were categorized as “outcomes” were further mapped according to whether they are clinician-reported (e.g., measurement of heart rate) or patient-reported (e.g., Euro-QoL 5D [18]). Afterwards, the outcome variables were mapped to categories of the World Health Organization's International Classification of Functioning, Disability and Health (ICF) [19]. The ICF reflects the broad spectrum of the lived experience of disease. It models health and functioning as the dynamic interaction between not only ‘Body Functions’ (the physiological and mental functions of body systems), ‘Body Structures’ (organs and limbs and other anatomical parts of the body, and ‘Health condition’ (disease, disorder or injury and can be classified using the International Classification of Diseases (ICD)), but also with ‘Activities & Participation’ (the ability and actual execution of a task by a person and his/her involvement in a life situation) [19,20]. This mapping was done by experts (BA, MC) using established ICF linking rules [21] (Example in S1 Table).

Descriptive analysis was carried out to describe the content and overlap of the variables contained in the NQRs and ICHOM-SS with numbers, frequencies and proportion.

## Results

**Fig 1:**
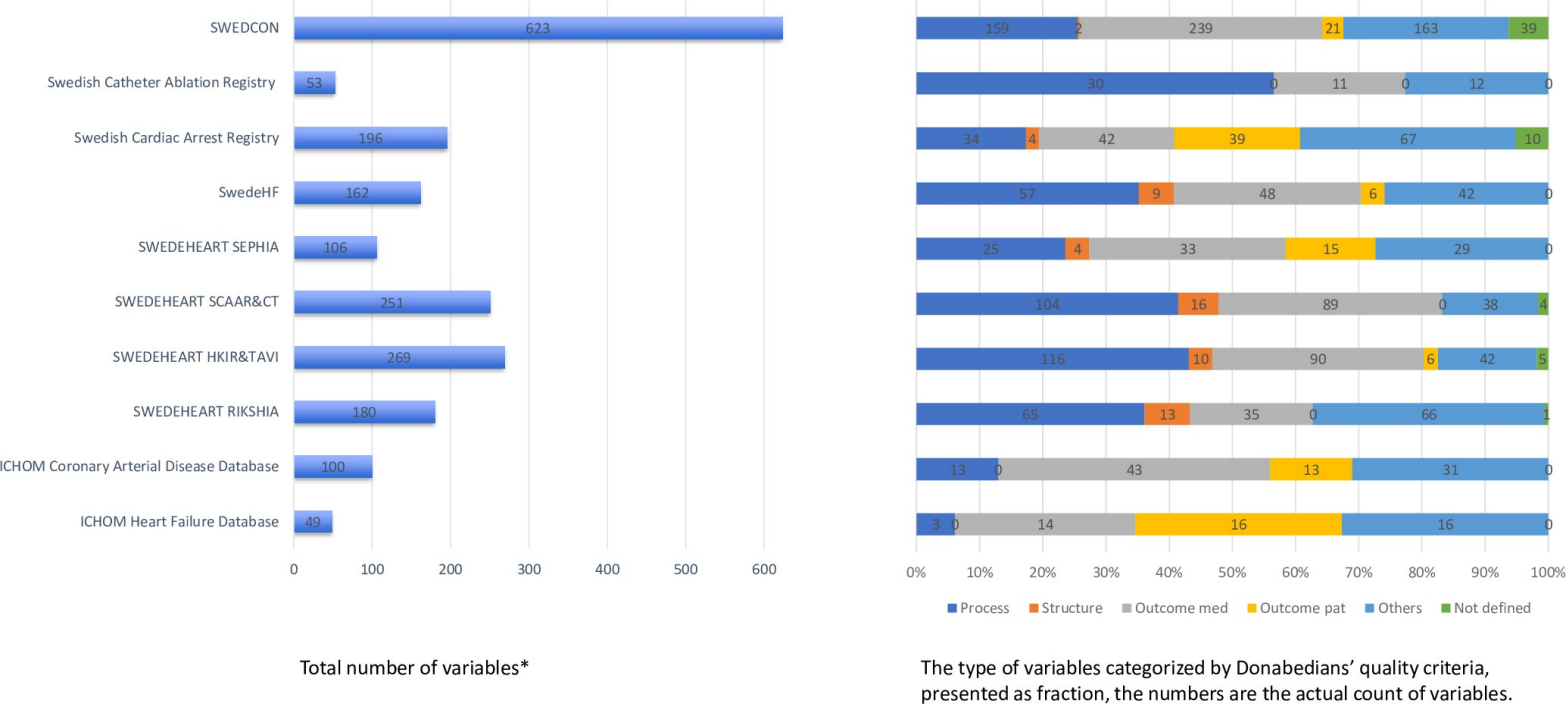
Overview of the total number of variables and the distribution (proportion in percentage) of the type of variables classified according to Donabedian’s quality criteria (*process*, *structures* and *outcomes*) in the Swedish National Quality Registries and the International Consortium of Health Outcomes Measurement (ICHOM) standard sets. SWEDEHEART = Swedish Websystem for Enhancement and Development of Evidence-based care in heart disease; RiksHIA = acute coronary care; SEPHIA = secondary prevention; HKIR & TAVI = heart surgery; SCAAR & CT = Coronary angiography and percutaneous coronary intervention; SWEDCON = Swedish Registry of Congenital Heart Disease; SwedeHF = Swedish Heart Failure Registry (available at www.kvalitetsregister.se). *Outcomes* were differentiated between information that were clinician-reported or patient-reported.”Others” were variables were administrative information like patient’s address, time point assessed or information regarding patient characteristics like patient’s age. There were some variables in the NQRs that had a variable code but no definition; these variables were categorized as “not defined”.

On average, each quality standard (NQR or ICHOM-SS) comprised at least 100 or more variables. SWEDCON was the most extensive NQR (N = 623) and included more than twice as many variables as the other NQRs. The Swedish Catheter Ablation Registry and ICHOM-SS for heart failure were the only two quality standards with around 50 variables each (Fig 1).

### Distribution of Donabedian’s quality criteria

In total, out of the 1989 variables addressed 606 process quality criteria (31%), 58 infrastructure quality criteria (3%) and 760 outcome quality criteria (38%). Five hundred and six variables were assigned as “others” (23%). Fifty nine variables were not defined (3%). Out of the 760 outcome variables, 644 variables were clinician-reported (85% of all outcomes). Compared to total number of variables in the quality standards, the ICHOM-SS for heart failure and coronary artery disease included the most outcome variables (61% and 56% respectively), followed by SEPHIA (45%), SWEDCON (42%), Swedish Cardiac Arrest Registry (41%). The Swedish Catheter Ablation Registry (57%) and HKIR & TAVI (43%) comprised mainly process variables (Fig 1).

### Distribution of the *Outcome* variables from a person-centered and biopsychosocial perspective of health

The quality standards encompassed mainly clinician-reported outcomes (range 19% in RiksHIA to 43% in ICHOM-SS for Coronary Arterial Disease) and less patient-reported outcomes (range 0% to 33%). The ICHOM-SS for heart failure and the Swedish Cardiac Arrest Registry included the most patient-reported outcomes compared to the total number of variables in each quality standard (33% and 20% respectively) (Fig 1).

**Fig 2:**
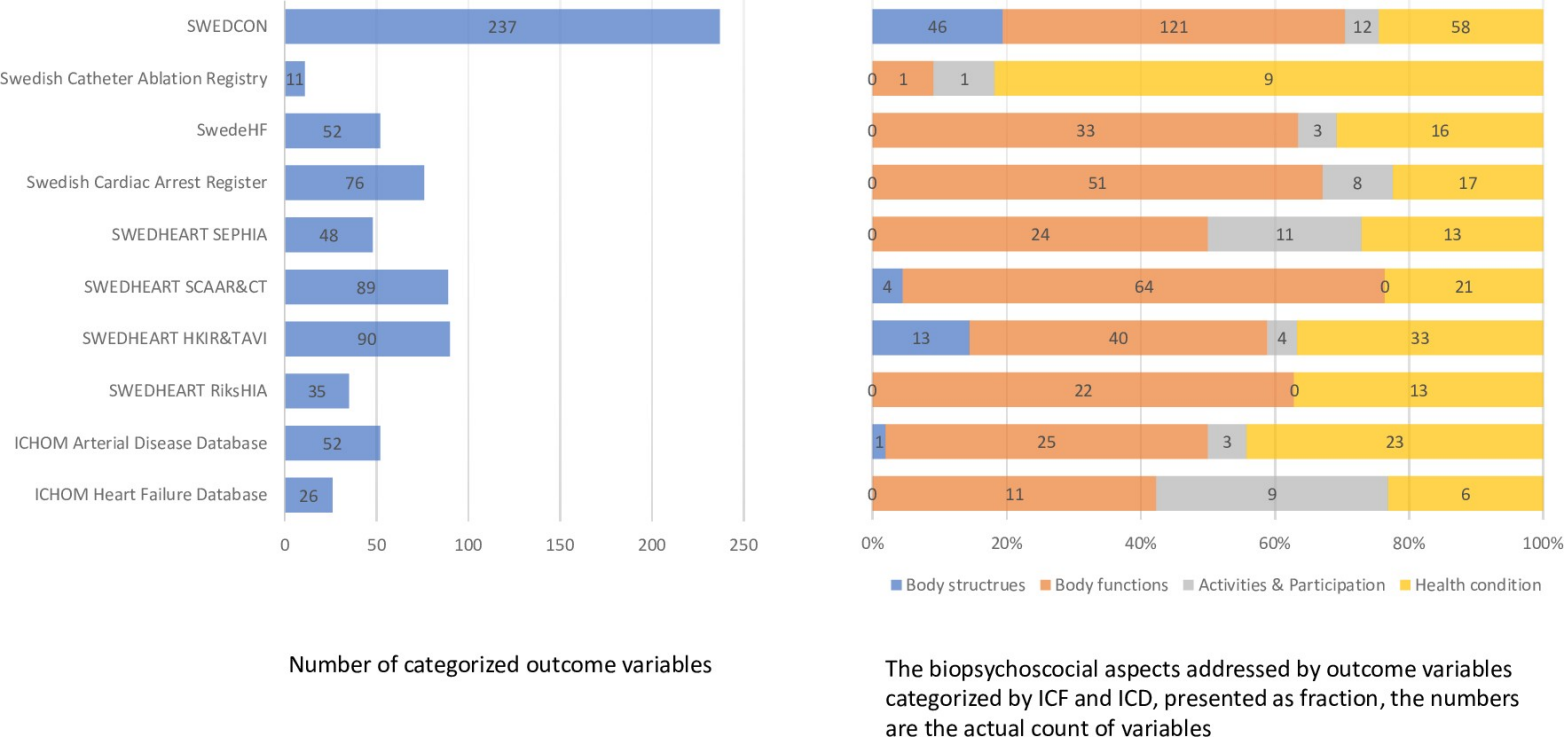
Overview of the total numbers of *outcome* variables and the distribution (proportion in percentage) of the type of variables according to ICF components and health condition. Body functions = physiological and mental functions of body systems; Body structures = organs, limbs and other anatomical parts; Activities & Participation = ability and actual execution of a task by a person; Health condition = Disease, disorder or injury as defined by the International Classification of Diseases (ICD).

Overall, the outcome variables addressing ‘Body functions’ (n = 392, 55%) were most common, followed by outcome variables addressing health conditions (n = 209, 29%); both were clinician-reported. ‘Body structures’ as well as aspects of ‘Activities & Participation’ were included at a much lower rate (n = 64, 9% and n = 51, 7% respectively). SWEDCON and SEPHIA comprised the most variables addressing ‘Activities & Participation’ (n = 12 and n = 11 respectively). The Swedish Catheter Ablation Registry included one variable mapped to ‘Body functions’, and one variable to ‘Activities & Participation’ (i.e. European Heart Rhythm Association (EHRA) score related to the impact of atrial fibrillation on daily activities), and nine variables related to health condition (Fig 2 and Table 2). Regarding ‘Activities & Participation’, self-care and mobility were common aspects while for example, generals tasks and demands as well as interpersonal interactions and relationships were neglected aspects. Almost all aspects on ‘Activities & Participation’ were patient-reported whereas aspects on ‘Body functions’ were mainly clinician-reported.

**Table 2:** Overview of outcome variables mapped to the ICF components of *Body functions*, *Body structures*, and *Activities & Participation*, stratified by patient- and clinician-reported. The numbers represent how many different aspects within each ICF chapter (e.g. b1, b2, b3, etc.) were addressed by the outcome variables (see more detailed information in the supplemental files S2 and S3). Darker colors indicate that 4 or more different aspects were addressed in the respective chapter. Note: Health conditions are not included here.

		Clinician-reported							Patient-reported (PROM)						
ICF Chapters		Swedish National Quality registries					ICHOM standard sets		Swedish National Quality registries					ICHOM standard sets	
Code	Title	SWEDCON	Catheter Ablation Registry	Cardiac arrest Registry	SwedeHF	SWEDEHEART	Coronary arterial disease	Heart Failure	SWEDCON	Catheter Ablation Registry	Cardiac arrest Registry	SwedeHF	SWEDEHEART	Coronary arterial disease	Heart Failure
**Body Functions**		** **	** **						** **						
**b1**	Mental functions	**0**	**0**	**2**	**1**	**0**	**0**	**0**	**2**	**0**	**5**	**1**	**1**	**2**	**3**
**b2**	Sensory functions and pain (pain)	**0**	**1**	**0**	**0**	**0**	**0**	**0**	**1**	**0**	**1**	**1**	**1**	**0**	**0**
**b3**	Voice and speech functions	**0**	**0**	**0**	**0**	**0**	**0**	**0**	**0**	**0**	**0**	**0**	**0**	**0**	**0**
**b4**	Functions of the cardiovascular, haematological, immunological and respiratory systems	**7**	**1**	**4**	**3**	**8**	**4**	**1**	**3**	**0**	**0**	**0**	**1**	**2**	**1**
**b5**	Functions of the digestive, metabolic and endocrine systems	**1**	**0**	**2**	**2**	**4**	**1**	**0**	**0**	**0**	**0**	**0**	**0**	**0**	**0**
**b6**	Genitourinary and reproductive functions	**1**	**0**	**1**	**1**	**1**	**1**	**1**	**0**	**0**	**0**	**0**	**0**	**0**	**0**
**b7**	Neuromusculoskeletal and movement-related functions	**2**	**0**	**0**	**0**	**0**	**0**	**0**	**0**	**0**	**0**	**0**	**0**	**0**	**0**
**b8**	Functions of the skin and related structures	**0**	**0**	**0**	**0**	**0**	**0**	**0**	**0**	**0**	**0**	**0**	**0**	**0**	**0**
**Body Structures**		** **	** **	** **	** **	** **	** **	** **	** **	** **	** **	** **	** **	** **	** **
**s1**	Structures of the nervous system	**1**	**0**	**0**	**0**	**1**	**0**	**0**	**0**	**0**	**0**	**0**	**0**	**0**	**0**
**s2**	Structure of brain	**0**	**0**	**0**	**0**	**0**	**0**	**0**	**0**	**0**	**0**	**0**	**0**	**0**	**0**
**s3**	Structures involved in voice and speech	**1**	**0**	**0**	**0**	**0**	**0**	**0**	**0**	**0**	**0**	**0**	**0**	**0**	**0**
**s4**	Structures of the cardiovascular, immunological and respiratory system	**2**	**0**	**0**	**0**	**2**	**0**	**0**	**0**	**0**	**0**	**0**	**0**	**0**	**0**
**s5**	Structures related to digestive, metabolic and endocrine systems	**0**	**0**	**0**	**0**	**0**	**0**	**0**	**0**	**0**	**0**	**0**	**0**	**0**	**0**
**s6**	Structures related to the genitourinary and reproductive functions	**0**	**0**	**0**	**0**	**0**	**0**	**0**	**0**	**0**	**0**	**0**	**0**	**0**	**0**
**s7**	Structures related to movement	**0**	**0**	**0**	**0**	**0**	**0**	**0**	**0**	**0**	**0**	**0**	**0**	**0**	**0**
**s8**	Skin and related structures	**0**	**0**	**0**	**0**	**0**	**0**	**0**	**0**	**0**	**0**	**0**	**0**	**0**	**0**
**Activities & Participation**		** **	** **	** **	** **	** **	** **	** **	** **	** **	** **	** **	** **	** **	** **
**d1**	Learning and applying knowledge	**0**	**0**	**0**	**0**	**0**	**0**	**0**	**0**	**0**	**0**	**0**	**0**	**0**	**0**
**d2**	General tasks and demands	**0**	**0**	**0**	**0**	**0**	**0**	**0**	**0**	**0**	**0**	**0**	**0**	**0**	**0**
**d3**	Communication	**0**	**0**	**0**	**0**	**0**	**0**	**0**	**0**	**0**	**0**	**0**	**0**	**0**	**0**
**d4**	Mobility	**0**	**0**	**0**	**0**	**0**	**0**	**0**	**1**	**0**	**1**	**1**	**1**	**1**	**2**
**d5**	Self-care	**0**	**0**	**0**	**1**	**0**	**0**	**0**	**1**	**0**	**1**	**1**	**1**	**0**	**3**
**d6**	Domestic life	**0**	**0**	**0**	**0**	**0**	**0**	**0**	**0**	**0**	**0**	**0**	**0**	**1**	**1**
**d7**	Interpersonal interactions and relationships	**0**	**0**	**0**	**0**	**0**	**0**	**0**	**0**	**0**	**0**	**0**	**0**	**0**	**0**
**d8**	Major life areas	**0**	**0**	**0**	**0**	**0**	**0**	**0**	**0**	**0**	**1**	**0**	**1**	**0**	**1**
**d9**	Community, social and civic life	**0**	**0**	**0**	**0**	**0**	**0**	**0**	**2**	**0**	**0**	**0**	**0**	**0**	**1**

Body functions = physiological and mental functions of body systems; Body structures = organs, limbs and other anatomical parts; Activities & Participation = ability and actual execution of a task by a person; Health condition = Disease, disorder or injury as defined by the International Classification of Diseases (ICD).

## Discussion

There is no doubt that quality standards and transparency are necessary for delivering high quality care. The results of our study revealed that individual quality standards differ in the volume of variables they contain ranging between 50 to 600 variables. About one third of registered information was about processes (i.e. treatment) and one third about outcomes (i.e. individuals’ functioning), while and approximately one quarter were administrative data. Most of the quality standard balanced the amount of process and outcome variables. However, some were more process-focused, e.g. Swedish Catheter Ablation Registry or RiksHIA, and others more outcome-focused, e.g. ICHOM Heart Failure Database and Coronary Arterial Disease Database, Swedish Cardiac Arrest Registry, or SWEDCON. While three quarters of all outcomes were clinician-reported, patient-reported outcomes were hardly documented. In fact, patient-reported outcomes represent only six percent of all the variables mapped, irrespective of Donabedian quality criteria. Most of the outcomes addressed Body functions or health conditions but rarely patients’ Activities & Participation.

Twenty years ago, the aforementioned IOM report emphasized the importance of person-centeredness and associating health care quality more closely to patients’ experiences” [22, p. 84]. In Berwick’s user’s manual for the IOM’s Quality Chasm Report, he outlined how to customize care according to individual patient needs, desires, and circumstances in order to achieve person-centeredness in health care [22]. However, two decades later, the investigated quality standards capture only limited information directly from patients. Nonetheless, Kamal et al. recently showed how enriched information on individual patient and family experiences as the fundamental outcome of interest could contribute to better outcomes, experience, value and science if integrated in registry-based learning health system, albeit for palliative care [23]. This is supported by Nelson and colleagues, who called for transforming registries into person-centered interactive learning systems that also enabled patients “to share their perceptions of health, function, and wellbeing with their care team in real time.” [24, p.4]. The registries would allow patients to select the measure that matters to them. Considering this, there is a need to modify the CVD quality standards analyzed in this study to include more patient-reported outcomes and outcomes that matter most to patients. For example, it might be important to include activities relevant to daily life, such as acquisition of goods and services, preparing meals, doing housework, remunerative employment, economic self-sufficiency, community life as well as recreation and leisure, family and intimate relationships. The latter named aspects are also commonly found in patient-reported outcome measures used in the cardiovascular field [25]. We can thus deduce that these aspects of functioning are valued by patients.

Moreover, the harmonization and a review of the quality standards could help to achieve a balance in the quantity of information available across Donabedian's quality criteria as well as in the quality of clinician-reported versus patient-reported information. This might reduce the burden of data collection and documentation on clinicians while also increasing quality standards’ value for quality improvement [10,11,26,27].

Then there is the issue of multimorbidity and redundant documentation. Multimorbidity is common in patients with cardiovascular disease [28]. For example, the CVDs heart failure, atrial fibrillation, and hypertension, are often accompanied by depression and chronic kidney disease [29]. Since several health conditions are involved, different registries may also become relevant for selected multimorbid patients. In other words, health professionals may be forced to document the same variables for these patients in several disease-specific registries, increasing the risk for double documentation. To complicate the situation, associating specific variables with the respective health condition may be difficult. For example, patients may have difficulties identifying whether particular activity restrictions and limitations are caused by atrial fibrillation or rather by hypertension or a combination of both health conditions. By harmonizing and creating a generic variable set common for CVD, information would be registered only once and irrespective of co-existing heart diseases.

The present study is limited by the fact that it is not a comprehensive overview of all existing national and international registries or quality standard sets in the field of cardiology. Instead, we chosen the registries and standard sets with freely accessible variable lists. Nevertheless, the results do give some insight in the content of quality standards (registries and standard sets) in the cardiovascular field and in the potential for increase their utility.

## Conclusions

Quality standards in cardiology generally focus on processes (e.g. treatment) and outcomes related to Body functions. Very little attention is given to patients’ lived experience of disease and their daily activities and participation. The study results can inform the steps toward harmonizing CVD-related data and serve as the starting point, for example to propose aspects of functioning, for the development of a person-centered quality indicator set.

## Supporting information

S1 Table Example mapping Swedish Heart Failure Registry (SwedeHF). (DOCX)

S2 Table Detailed overview of outcome variables mapped to ICF’s *Body functions* and ICF’s *Body structures*, stratified by patient- or clinician-reported. C = clinician-reported, P = patient-reported, Y = Yes, aspects of this chapter are included; x = this specific aspect is included.
(DOCX)

S3 Table Detailed overview of outcome variables mapped to ICF’s *Activities & Participation*, stratified by patient- or clinician-reported. C = clinician-reported, P = patient-reported; Y = Yes, aspects of this chapter are included; x = this specific aspect is included.
(DOCX)
