# Correction: Content comparison and person-centeredness of standards for quality improvement in cardiovascular health care

**DOI:** 10.1371/journal.pone.0251226

**Published:** 2021-04-29

**Authors:** Beatrix Algurén, Tomas Jernberg, Peter Vasko, Melissa Selb, Michaela Coenen

There is an error in affiliation 5 for authors Melissa Selb and Michaela Coenen. The correct affiliation 5 is: ICF Research Branch, Nottwil, Switzerland.

The ICF Research Branch is no longer a cooperation partner of the WHO Collaborating Centre for the Family of International Classifications (at DIMDI).
