# Supplementary material for: Content comparison and person-centeredness of standards for quality improvement in cardiovascular health care
Source: PLoS One. 2021 Jan 7;16(1):e0244874. doi: 10.1371/journal.pone.0244874 (PMC7790275; doi:10.1371/journal.pone.0244874)
Supplement: S1 Table — (DOCX) [file pone.0244874.s001.docx]

S1 Table. Example mapping Swedish Heart Failure Registry (SwedeHF).

| **Nr.** | **Name of Form** | **Variable as appeared** | **Variable as appeared in the register - English** | **Definition of variable as appeared** | **Definition of variable as appeared** | **Quality** | **Specification of others** | **ICF code 1_1** |
| --- | --- | --- | --- | --- | --- | --- | --- | --- |
|  |  | **in the register - Swedish** |  | **in the register - Swedish** | **in the register - English** |  |  |  |
| The number of the variable |  |  |  |  |  | Structure | Administrative issue |  |
| as it appears in the register |  |  |  |  |  | Process | Diagnosis-related information |  |
|  |  |  |  |  |  | Outcome - patient | Patient characteristic |  |
|  |  |  |  |  |  | Outcome - medical / HP |  |  |
|  |  |  |  |  |  |  | Others |  |
|  |  |  |  |  |  | Others |  |  |
|  |  |  |  |  |  |  |  |  |
| 1 | RiksSvikt | A2_INHIBITORS_AMOUNT | A2_INHIBITORS_AMOUNT | A2 dos | A2 Inhibitors amount | Process |  |  |
| 2 | RiksSvikt | A2_INHIBITORS_TYPE | A2_INHIBITORS_TYPE | A2 generika | A2 Inhibitors type | Process |  |  |
| 3 | RiksSvikt | A2_INHIBITORS | A2_INHIBITORS | A2-blockerare/ARB | A2 inhibitors | Process |  |  |
| 4 | RiksSvikt | DOSA2 | DOSA2 | A2-blockerare/ARB | A2 blocker | Process |  |  |
| 5 | RiksSvikt | ACE_INHIBITORS_AMOUNT | ACE_INHIBITORS_AMOUNT | ACE dos | ACE dose | Process |  |  |
| 6 | RiksSvikt | ACE_INHIBITORS_TYPE | ACE_INHIBITORS_TYPE | ACE generika | ACE generic | Process |  |  |
| 7 | RiksSvikt | ACE_INHIBITORS | ACE_INHIBITORS | ACE-hämmare | ACE Inhibitors | Process |  |  |
| 8 | RiksSvikt | BEFREG_ADDRESS | BEFREG_ADDRESS | Adress vid senaste kontroll | Adresses with the last control | Others | Patient characteristics |  |
| 9 | RiksSvikt | ACTIVITIES | ACTIVITIES | Aktiviteter | Activities | Outcome - patient |  | d |
| 10 | RiksSvikt | ALDOSTERONANTAGONIST | ALDOSTERONANTAGONIST | Aldosteronantagonist | Aldosteron-antagonis | Process |  |  |
| 11 | RiksSvikt | ALKOHOL | ALKOHOL | Alkohol | Alcohol | Others | Patient characteristics |  |
| 12 | RiksSvikt | AMIODARONE | AMIODARONE | Amiodarone | Amiodarone | Process |  |  |
| 13 | RiksSvikt | OUTOFBREATH | OUTOFBREATH | Andfåddhet | out of breath | Outcome - medical / HP |  | b4551 |
| 14 | RiksSvikt | ANTOVRLAKEM | ANTOVRLAKEM | Antal övriga läkemedel | Amount of drugs | Process |  |  |
| 15 | RiksSvikt | ANTIKOAGULANTIA | ANTIKOAGULANTIA | Antikoagulantia | Antikoagulantia | Process |  |  |
| 16 | RiksSvikt | ORAL_ANTICOAGULANTS | ORAL_ANTICOAGULANTS | Antikoagulantia | oral anticoagulants | Process |  |  |
| 17 | RiksSvikt | APOA1 | APOA1 | APOA1 | APOA 1 | Outcome - medical / HP |  | b5403 |
| 18 | RiksSvikt | APOB | APOB | APOB | APOB | Outcome - medical / HP |  | b5403 |
| 19 | RiksSvikt | ARBETSPROV | ARBETSPROV | Arbetsprov | Work sample | Process |  |  |
| 20 | RiksSvikt | ASATRC | ASATRC | ASA/övr. trombocythämmare | ASA / Trombocyt inhibitor | Process |  |  |
| 21 | RiksSvikt | DEATH | DEATH | Avliden | death | Outcome - medical / HP |  | hc |
| 22 | RiksSvikt | AVLIDEN | DECEASED | Avliden under vårdtillfället | Death during the treatment | Outcome - medical / HP |  | hc |
| 23 | RiksSvikt | HB | HB | B-HB | Haemoglobin | Outcome - medical / HP |  | b540 |
| 24 | RiksSvikt | HBA1C | HBA1C | B-HbA1c | HbA1c | Outcome - medical / HP |  | b540 |
| 25 | RiksSvikt | HBA1CMOL | HBA1CMOL | B-HbA1c mmol/mol | HbA1c mmol/mol | Outcome - medical / HP |  | b540 |
| 26 | RiksSvikt | BETA_INHIBITORS_AMOUNT | BETA_INHIBITORS_AMOUNT | beta dos | beta blocker amount | Process |  |  |
| 27 | RiksSvikt | BETA_INHIBITORS | BETA_INHIBITORS | Betablockerare | beta blocker | Process |  |  |
| 28 | RiksSvikt | BETA_INHIBITORS_TYPE | BETA_INHIBITORS_TYPE | Betablockerare generika | beta blocker type | Process |  |  |
| 29 | RiksSvikt | BLODSOCKER | BLODSOCKER | Blodsocker | Blood sugar | Outcome - medical / HP |  | b5401 |
| 30 | RiksSvikt | BOFORM | ACCOMODATION FORM | Boendeform | BOFORM | Others | Patient characteristics |  |
| 31 | RiksSvikt | BTDIASTOLISKT | BTDIASTOLISKT | BT-diastoliskt | BT-diastolic | Outcome - medical / HP |  | b420 |
| 32 | RiksSvikt | BTSYSTOLISKT | BTSYSTOLISKT | BT-systoliskt | BT-sistolic | Outcome - medical / HP |  | b420 |
| 33 | RiksSvikt | BTDIASTOLISKT_MISS | BTDIASTOLISKT_MISS | BTDIASTOLISKT_MISS | BT-diastolic_miss | Outcome - medical / HP |  | b420 |
| 34 | RiksSvikt | BTSYSTOLISKT_MISS | BTSYSTOLISKT_MISS | BTSYSTOLISKT_MISS | BT-sistolic_miss | Outcome - medical / HP |  | b420 |
| 35 | RiksSvikt | CENTREID | CENTREID | CENTREID | centre ID | Structure |  |  |
| 36 | RiksSvikt | CIVILSTATUS | CIVILSTATUS | Civilstånd | Civil status | Others | Patient characteristics |  |
| 37 | RiksSvikt | CREATEDBY | CREATEDBY | CREATEDBY | created by | Structure |  |  |
| 38 | RiksSvikt | CREATEDDATE | CREATEDDATE | CREATEDDATE | created date | Others | Administrative issue |  |
| 39 | RiksSvikt | BASEDATE | BASEDATE | Datum för patientens första reg | Date for patient at the first registration | Others | Administrative issue |  |
| 40 | RiksSvikt | EKGDATE | EKGDATE | Datum för senast gjorda EKG | Date for last EKG | Others | Administrative issue |  |
| 41 | RiksSvikt | EKODATE | EKODATE | Datum för senast gjorda EKO | Date for last EKO | Others | Administrative issue |  |
| 42 | RiksSvikt | FOLLOWUP_DATE | FOLLOWUP_DATE | Datum för uppföljning | follow up date | Others | Administrative issue |  |
| 43 | RiksSvikt | SUMMONS1_SENT_DATE | SUMMONS1_SENT_DATE | Datum för utskick 1 | Sending Date 1 | Others | Administrative issue |  |
| 44 | RiksSvikt | SUMMONS2_SENT_DATE | SUMMONS2_SENT_DATE | Datum för utskick 2 | Sending Date 2 | Others | Administrative issue |  |
