# Supplementary material for: Content comparison and person-centeredness of standards for quality improvement in cardiovascular health care
Source: PLoS One. 2021 Jan 7;16(1):e0244874. doi: 10.1371/journal.pone.0244874 (PMC7790275; doi:10.1371/journal.pone.0244874)
Supplement: S2 Table — C = clinician-reported, P = patient-reported, Y = Yes, aspects of this chapter are included; x = this specific aspect is included. (DOCX) [file pone.0244874.s002.docx]

S2 Table1. Detailed overview of outcome variables mapped to ICF’s *Body functions* and ICF’s *Body structures*, stratified by patient- or clinician reported.

| **ICF categories** | | **Sum of NQRs and ICHOM-SS (n)** | | | **Swedish National Quality registries** | | | | | | | | | | **ICHOM standard sets** | | | |
| --- | --- | --- | --- | --- | --- | --- | --- | --- | --- | --- | --- | --- | --- | --- | --- | --- | --- | --- |
| **Code** | **Title** |  |  |  | **SWEDCON** | | **Catheter Ablation Registry** | | **Cardiac arrest Registry** | | **SwedeHF** | | **SWEDEHEART** | | **coronary arterial disease** | | **Heart Failure** | |
|  |  | **C** | **P** |  | **C** | **P** | **C** | **P** | **C** | **P** | **C** | **P** | **C** | **P** | **C** | **P** | **C** | **P** |
| **Body Functions** | |  |  |  |  |  |  |  |  |  |  |  |  |  |  |  |  |  |
| **b1** | **Mental functions** | **2** | **6** |  |  | **Y** |  |  | **Y** | **Y** | **Y** | **Y** |  | **Y** |  | **Y** |  | **Y** |
| b110 | Consciousness functions | 0 | 0 |  |  |  |  |  | x |  |  |  |  |  |  |  |  |  |
| b114 | Orientation functions | 1 | 0 |  |  |  |  |  |  |  |  |  |  |  |  |  |  |  |
| b126 | Temperament and personality functions | 0 | 1 |  |  |  |  |  |  | x |  |  |  |  |  |  |  |  |
| b130 | Energy and drive functions | 1 | 4 |  |  | x |  |  |  | x | x |  |  |  |  | x |  | x |
| b134 | Sleep functions | 0 | 1 |  |  |  |  |  |  |  |  |  |  |  |  |  |  | x |
| b140 | Attention functions | 1 | 0 |  |  |  |  |  | x |  |  |  |  |  |  |  |  |  |
| b144 | Memory functions | 0 | 0 |  |  |  |  |  |  |  |  |  |  |  |  |  |  |  |
| b147 | Psychomotor functions | 0 | 1 |  |  |  |  |  |  | x |  |  |  |  |  |  |  |  |
| b152 | Emotional functions | 0 | 6 |  |  | x |  |  |  | x |  | x |  | x |  | x |  | x |
| b160 | Thought functions (content) | 0 | 1 |  |  |  |  |  |  | x |  |  |  |  |  |  |  |  |
| **b2** | **Sensory functions and pain** | **1** | **4** |  |  | **Y** | **Y** |  |  | **Y** |  | **Y** |  | **Y** |  |  |  |  |
| b260 | Proprioceptive function | 0 | 0 |  |  |  |  |  |  |  |  |  |  |  |  |  |  |  |
| b280 | Sensation of pain | 1 | 4 |  |  | x | x |  |  | x |  | x |  | x |  |  |  |  |
| **b3** | **Voice and speech functions** | **0** | **0** |  |  |  |  |  |  |  |  |  |  |  |  |  |  |  |
| b310 | Voice functions | 0 | 0 |  |  |  |  |  |  |  |  |  |  |  |  |  |  |  |
| **b4** | **Functions of the cardiovascular, haematological, immunological and respiratory systems** | **7** | **4** |  |  | **Y** | **Y** |  | **Y** |  | **Y** |  | **Y** | **Y** | **Y** | **Y** | **Y** | **Y** |
| b410 | Heart functions | 6 | 0 |  | x |  |  |  | x |  | x |  | x |  | x |  | x |  |
| b415 | Blood vessel functions | 4 | 1 |  | x |  | x |  | x |  |  |  | x |  |  |  |  | x |
| b420 | Blood pressure functions | 5 | 0 |  | x |  |  |  | x |  | x |  | x |  | x |  |  |  |
| b430 | Haematological system functions | 2 | 0 |  | x |  |  |  |  |  |  |  | x |  |  |  |  |  |
| b435 | Immunological system functions | 1 | 0 |  | x |  |  |  |  |  |  |  | x |  | x |  |  |  |
| b440 | Respiration functions | 2 | 1 |  |  | x |  |  | x |  |  |  |  |  | x |  |  |  |
| b445 | Respiratory muscle functions | 0 | 0 |  |  |  |  |  |  |  |  |  |  |  |  |  |  |  |
| b450 | Additional respiratory functions | 0 | 0 |  |  |  |  |  |  |  |  |  |  |  |  |  |  |  |
| b455 | Exercise tolerance functions | 3 | 2 |  | x | x |  |  |  |  | x |  | x |  |  | x |  |  |
| b460 | Sensations associated with cardiovascular and respiratory functions | 2 | 3 |  | x | x |  |  |  |  |  |  | x | x |  | x |  |  |
| b469 | Additional functions and sensations of the cardiovascular and respiratory systems, other specified and unspecified | 1 | 0 |  |  |  |  |  |  |  |  |  | x |  |  |  |  |  |
| **b5** | **Functions oft he digestive, metabolic and endocrine systems** | **5** | **0** |  | **Y** |  |  |  | **Y** |  | **Y** |  | **Y** |  | **Y** |  |  |  |
| b510 | Ingestion functions | 0 | 0 |  |  |  |  |  |  |  |  |  |  |  |  |  |  |  |
| b515 | Digestive functions | 1 | 0 |  |  |  |  |  |  |  |  |  | x |  |  |  |  |  |
| b520 | Assimilation functions | 2 | 0 |  |  |  |  |  | x |  |  |  | x |  |  |  |  |  |
| b525 | Defecation functions | 0 | 0 |  |  |  |  |  |  |  |  |  |  |  |  |  |  |  |
| b530 | Weight maintenance functions | 1 | 0 |  |  |  |  |  |  |  |  |  | x |  |  |  |  |  |
| b540 | General metabolic functions | 3 | 0 |  |  |  |  |  |  |  | x |  | x |  | x |  |  |  |
| b545 | Water, mineral and electrolyte balance functions | 0 | 0 |  |  |  |  |  |  |  | x |  |  |  |  |  |  |  |
| b550 | Thermoregulatory functions | 2 | 0 |  | x |  |  |  | x |  |  |  |  |  |  |  |  |  |
| **b6** | **Genitourinary and reproductive functions** | **6** | **0** |  | **Y** |  |  |  | **Y** |  | **Y** |  | **Y** |  | **Y** |  | **Y** |  |
| b610 | Urinary excretory functions | 6 | 0 |  | x |  |  |  | x |  | x |  | x |  | x |  | x |  |
| b620 | Urination functions | 0 | 0 |  |  |  |  |  |  |  |  |  |  |  |  |  |  |  |
| b640 | Sexual functions | 0 | 0 |  |  |  |  |  |  |  |  |  |  |  |  |  |  |  |
| **b7** | **Neuromusculoskeletal and movement-related functions** | **1** | **0** |  | **Y** |  |  |  |  |  |  |  |  |  |  |  |  |  |
| b710 | Mobility of joint functions | 0 | 0 |  |  |  |  |  |  |  |  |  |  |  |  |  |  |  |
| b730 | Muscle power functions | 0 | 0 |  |  |  |  |  |  |  |  |  |  |  |  |  |  |  |
| b735 | Muscle tone functions | 1 | 0 |  | x |  |  |  |  |  |  |  |  |  |  |  |  |  |
| b740 | Muscle endurance functions | 0 | 0 |  |  |  |  |  |  |  |  |  |  |  |  |  |  |  |
| b760 | Control of voluntary movement functions | 1 | 0 |  | x |  |  |  |  |  |  |  |  |  |  |  |  |  |
| b780 | Sensations related to muscles and movement functions | 0 | 0 |  |  |  |  |  |  |  |  |  |  |  |  |  |  |  |
| **b8** | **Functions of the skin and related structures** | **0** | **0** |  |  |  |  |  |  |  |  |  |  |  |  |  |  |  |
| b810 | Protective functions of the skin | 0 | 0 |  |  |  |  |  |  |  |  |  |  |  |  |  |  |  |
| b820 | Repair functions of the skin | 0 | 0 |  |  |  |  |  |  |  |  |  |  |  |  |  |  |  |
|  |  |  |  |  |  |  |  |  |  |  |  |  |  |  |  |  |  |  |
| **Body structures** | |  |  |  |  |  |  |  |  |  |  |  |  |  |  |  |  |  |
| **s1** | **Structures of the nervous system** | **2** | **0** |  | **Y** |  |  |  |  |  |  |  | **Y** |  |  |  |  |  |
| s110 | Structure of brain | 1 | 0 |  |  |  |  |  |  |  |  |  | x |  |  |  |  |  |
| **s3** | **Structures involved in voice and speech** | **1** | **0** |  | **Y** |  |  |  |  |  |  |  |  |  |  |  |  |  |
| **s4** | **Structures oft he cardiovascular, immunological and respiratory system** | **2** | **0** |  | **Y** |  |  |  |  |  |  |  | **Y** |  |  |  |  |  |
| s410 | Structure of cardiovascular system | 2 | 0 |  | x |  |  |  |  |  |  |  | x |  |  |  |  |  |
| s430 | Structure of respiratory system | 2 | 0 |  | x |  |  |  |  |  |  |  | x |  |  |  |  |  |
| **s7** | **Structures related to movement** | **0** | **0** |  |  |  |  |  |  |  |  |  |  |  |  |  |  |  |
| s760 | Structure of trunk | 0 | 0 |  |  |  |  |  |  |  |  |  |  |  |  |  |  |  |
| **s8** | **Skin and related structures** | **0** | **0** |  |  |  |  |  |  |  |  |  |  |  |  |  |  |  |
| s810 | Structure of areas of skin | 0 | 0 |  |  |  |  |  |  |  |  |  |  |  |  |  |  |  |

C= clinician-reported, P= patient-reported
