# Supplementary material for: Content comparison and person-centeredness of standards for quality improvement in cardiovascular health care
Source: PLoS One. 2021 Jan 7;16(1):e0244874. doi: 10.1371/journal.pone.0244874 (PMC7790275; doi:10.1371/journal.pone.0244874)
Supplement: S3 Table — C = clinician-reported, P = patient-reported; Y = Yes, aspects of this chapter are included; x = this specific aspect is included. (DOCX) [file pone.0244874.s003.docx]

S3 Table. Detailed overview of outcome variables mapped to ICF’s *Activities & Participation*, stratified by patient- or clinician-reported.

| **ICF categories** | | | **Sum of NQRs and ICHOM-SS (n)** | | | **Swedish National Quality registries** | | | | | | | | | | **ICHOM standard sets** | | | |
| --- | --- | --- | --- | --- | --- | --- | --- | --- | --- | --- | --- | --- | --- | --- | --- | --- | --- | --- | --- |
| **Code** | **Title** | |  |  |  | **SWEDCON** | | **Catheter Ablation Registry** | | **Cardiac arrest Registry** | | **SwedeHF** | | **SWEDEHEART** | | **Coronary arterial disease** | | **Heart Failure** | |
|  |  | | **C** | **P** |  | **C** | **P** | **C** | **P** | **C** | **P** | **C** | **P** | **C** | **P** | **C** | **P** | **C** | **P** |
| **Activities & Participation** | | |  |  |  |  |  |  |  |  |  |  |  |  |  |  |  |  |  |
| **d1** | | **Learning and applying knowledge** |  |  |  |  |  |  |  |  |  |  |  |  |  |  |  |  |  |
| d155 | | Acquiring skills | 0 | 0 |  |  |  |  |  |  |  |  |  |  |  |  |  |  |  |
| d177 | | Making decisions | 0 | 0 |  |  |  |  |  |  |  |  |  |  |  |  |  |  |  |
| **d2** | | **General tasks and demands** | **0** | **0** |  |  |  |  |  |  |  |  |  |  |  |  |  |  |  |
| d230 | | Carrying out daily routine | 0 | 0 |  |  |  |  |  |  |  |  |  |  |  |  |  |  |  |
| d240 | | Handling stress and other psychological demands | 0 | 0 |  |  |  |  |  |  |  |  |  |  |  |  |  |  |  |
| **d3** | | **Communication** | **0** | **0** |  |  |  |  |  |  |  |  |  |  |  |  |  |  |  |
| d330 | | Speaking | 0 | 0 |  |  |  |  |  |  |  |  |  |  |  |  |  |  |  |
| **d4** | | **Mobility** | **0** | **6** |  |  | **Y** |  |  |  | **Y** |  | **Y** |  | **Y** |  | **Y** |  | **Y** |
| d410 | | Changing basic body position | 0 | 0 |  |  |  |  |  |  |  |  |  |  |  |  |  |  |  |
| d415 | | Maintaining a body position | 0 | 0 |  |  |  |  |  |  |  |  |  |  |  |  |  |  |  |
| d420 | | Transferring oneself | 0 | 0 |  |  |  |  |  |  |  |  |  |  |  |  |  |  |  |
| d430 | | Lifting and carrying objects | 0 | 1 |  |  |  |  |  |  |  |  |  |  |  |  | x |  |  |
| d440 | | Fine hand use | 0 | 0 |  |  |  |  |  |  |  |  |  |  |  |  |  |  |  |
| d445 | | Hand and arm use | 0 | 0 |  |  |  |  |  |  |  |  |  |  |  |  |  |  |  |
| d450 | | Walking | 0 | 1 |  |  |  |  |  |  |  |  |  |  |  |  |  |  | x |
| d455 | | Moving around | 0 | 1 |  |  |  |  |  |  |  |  |  |  |  |  |  |  | x |
| d460 | | Moving around in different locations | 0 | 0 |  |  |  |  |  |  |  |  |  |  |  |  |  |  |  |
| d465 | | Moving around using equipment | 0 | 1 |  |  | x |  |  |  |  |  |  |  |  |  |  |  |  |
| d470 | | Using transportation | 0 | 0 |  |  |  |  |  |  |  |  |  |  |  |  |  |  |  |
| d475 | | Driving | 0 | 0 |  |  |  |  |  |  |  |  |  |  |  |  |  |  |  |
| d480 | | Riding animals for transportation | 0 | 0 |  |  |  |  |  |  |  |  |  |  |  |  |  |  |  |
| **d5** | | **Self-care** | **1** | **5** |  |  | **Y** |  |  |  | **Y** | **Y** | **Y** |  | **Y** |  |  |  | **Y** |
| d510 | | Washing oneself | 0 | 1 |  |  |  |  |  |  |  |  |  |  |  |  |  |  | x |
| d520 | | Caring for body parts | 0 | 0 |  |  |  |  |  |  |  |  |  |  |  |  |  |  |  |
| d530 | | Toileting | 0 | 0 |  |  |  |  |  |  |  |  |  |  |  |  |  |  |  |
| d540 | | Dressing | 0 | 0 |  |  |  |  |  |  |  |  |  |  |  |  |  |  |  |
| d550 | | Eating | 0 | 0 |  |  |  |  |  |  |  |  |  |  |  |  |  |  |  |
| d560 | | Drinking | 0 | 0 |  |  |  |  |  |  |  |  |  |  |  |  |  |  |  |
| d570 | | Looking after one’s health | 0 | 3 |  |  | x |  |  |  |  |  |  |  | x |  |  |  | x |
| d599 | | Self-care, unspecified | 0 | 1 |  |  |  |  |  |  |  |  |  |  |  |  |  |  | x |
| **d6** | | **Domestic life** | **0** | **2** |  |  |  |  |  |  |  |  |  |  |  |  | **Y** |  | **Y** |
| d620 | | Acquisition of goods and services | 0 | 0 |  |  |  |  |  |  |  |  |  |  |  |  |  |  |  |
| d630 | | Preparing meals | 0 | 0 |  |  |  |  |  |  |  |  |  |  |  |  |  |  |  |
| d640 | | Doing housework | 0 | 0 |  |  |  |  |  |  |  |  |  |  |  |  |  |  |  |
| **d7** | | **Interpersonal interactions and relationships** | **0** | **0** |  |  |  |  |  |  |  |  |  |  |  |  |  |  |  |
| d760 | | Family relationships | 0 | 0 |  |  |  |  |  |  |  |  |  |  |  |  |  |  |  |
| d770 | | Intimate relationships | 0 | 0 |  |  |  |  |  |  |  |  |  |  |  |  |  |  |  |
| **d8** | | **Major life areas** | **0** | **3** |  |  |  |  |  |  | **Y** |  |  |  | **Y** |  |  |  | **Y** |
| d850 | | Remunerative employment | 0 | 3 |  |  |  |  |  |  | x |  |  |  | x |  |  |  | x |
| d870 | | Economic self-sufficiency | 0 | 0 |  |  |  |  |  |  |  |  |  |  |  |  |  |  |  |
| **d9** | | **Community, social and civic life** | **0** | **2** |  |  | **Y** |  |  |  |  |  |  |  |  |  |  |  | **Y** |
| d910 | | Community life | 0 | 1 |  |  | x |  |  |  |  |  |  |  |  |  |  |  |  |
| d920 | | Recreation and leisure | 0 | 2 |  |  | x |  |  |  |  |  |  |  |  |  |  |  | x |

C= clinician-reported, P= patient-reported
